# Difference in the Inhibitory Effect of Temozolomide on TJ905 Glioma Cells and Stem Cells

**DOI:** 10.3389/fneur.2017.00474

**Published:** 2017-09-13

**Authors:** Feng Jin, Guang-Kui Han, Hao Zhang, Ran Zhang, Gen-Hua Li, Song Feng, Xian-Yun Qin, Ling-Sheng Kong, Quan-Min Nie, Hua-Rong Li, Lei Zhao

**Affiliations:** ^1^Department of Neurosurgery, Affiliated Hospital of Jining Medical University, Shandong Provincial Key Laboratory of Stem Cells and Neuro-Oncology, Jining, China; ^2^Department of Integrated Traditional Chinese and Western Medicine, Union Hospital, Tongji Medical College, Huazhong University of Science and Technology, Wuhan, China; ^3^Department of Infectious Diseases, Union Hospital, Tongji Medical College, Huazhong University of Science and Technology, Wuhan, China

**Keywords:** Livin, temozolomide, glioma, cancer stem cells, proliferation

## Abstract

This study aims to determine the difference in the inhibitory effect of temozolomide (TMZ) on TJ905 glioma cells and stem cells. TJ905 cancer stem cells were isolated. Livin is a member of the inhibitor of apoptosis protein family. The TJ905 cells and cancer stem cells were transfected with a Livin-shRNA and negative-shRNA, respectively, and then treated with TMZ. At 48 h post-transfection, a cell counting kit 8 assay, flow cytometry, and real-time qPCR were performed to detect cell proliferation, the cell cycle, and the expression of the Caspase-3, -7, and -9 mRNAs, respectively. As a result, the suppressive effect of TMZ on TJ905 cells was more significant than its effect on TJ905 cancer stem cells. TMZ exerted an inhibitory effect on the growth of TJ905 glioma cells by arresting them at G0/G1 phase and arresting cancer stem cells at S phase in a dose-dependent manner. TMZ inhibited Livin mRNA expression and increased the expression of the Caspase-3, -7, and -9 mRNAs. Low Livin mRNA expression induced high levels of Caspase-3, -7, and -9 expressions, thus promoting the apoptosis of both TJ905 cells and cancer stem cells in response to TMZ treatment. The TJ905 cells transfected with the Livin-shRNA were more sensitive to TMZ, whereas the TJ905 glioma stem cells transfected with the Livin-shRNA showed no significant changes in their sensitivity to TMZ. In conclusion, the Livin gene may play an important role in the resistance mechanisms of TJ905 glioma cells and cancer stem cells. However, Livin had a more distinct role in TMZ resistance, cell proliferation, and the cell cycle in TJ905 glioma cells than in cancer stem cells.

## Introduction

Glioma is the most common intracranial malignant tumor. Although surgery, radiation, and chemotherapy have achieved great progress, glioma is still difficult to completely cure (1). The challenge lies in recurrence of the tumor and drug resistance to chemotherapy. Therefore, studies identifying new methods and ideas that overcome the limitations of current therapies and prolong the survival of patients suffering from gliomas are required (2).

In recent years, studies of cancer stem cells have highlighted their roles in tumorigenesis and the development of cancer drug resistance (3). The theory postulates that cancer stem cells display natural chemoresistance, a relatively stationary state, the capacity to repair damaged DNA, high expression of ABC transporters, and a strong ability to resist apoptosis; thus, these cells represent a new research target for chemotherapeutic drug resistance in glioma (4). Cancer stem cells may be the key to solve the problems of chemotherapy resistance and tumor relapse (5).

Livin, which was identified as a new member of the inhibitor of apoptosis protein (IAP) family, is the only member of the eight member IAP family that consists of two subunits. The alpha and beta subunits are likely to facilitate the stronger anti-apoptotic activity of Livin. As shown in our previous studies, Livin is associated with the resistance of glioma cells and cancer stem cells to chemotherapeutic drugs (6, 7).

Temozolomide (TMZ), a type of alkylating reagent, is widely used to treat high-grade glioma (8). Based on findings from recent clinical studies, TMZ prolongs the average median survival time, thereby becoming the first-line chemotherapy drug for gliomas when combined with radiation (9). Patients with glioblastoma containing a methylated MGMT promoter can benefit from TMZ (10). However, the mechanism by which Livin influences the proliferation of glioma cells and stem cells treated with chemotherapeutic agents remains unclear. This study aims to explore the role of the anti-apoptotic gene Livin in the effect of TMZ on the proliferation and drug resistance of glioma stem cells, offering a new idea to solve drug resistance in glioma.

## Materials and Methods

### Chemicals and Reagents

Dulbecco’s Modified Eagle’s Medium (DMEM) and fetal bovine serum (FBS) were obtained from HyClone (Logan, UT, USA). Epidermal growth factor, basic fibroblast growth factor (bFGF), and leukemia inhibitory factor (LIF) were obtained from Peprotech (Rocky Hill, NJ, USA). The B27 (1×) serum-free supplement was purchased from Gibco Life Technologies (Grand Island, NY, USA). Nestin, GFAP, and β-tubulin were obtained from Santa Cruz Biotechnology (Santa Cruz, CA, USA). FITC-conjugated IgG and related secondary antibodies were obtained from Boshide Co., Ltd. (Wuhan, China). The anti-CD133 antibody, its buffer solution and the CD133 cell isolation kit (magnetic activated cell sorting method) were purchased from Miltenyi Biotec GmbH (Bergisch Gladbach, Germany). The cell counting kit 8 (CCK-8) was obtained from Dojindo Molecular Technologies, Inc. (Kumamoto, Japan). SYBR Green I fluorochrome was purchased from Biotium, Inc. (Hayward, CA, USA).

### Main Instruments

Microplate reader (model: 1510) and the carbon dioxide incubator were obtained from Thermo Fisher (MA, USA). The inverted microscope (model: OLYMPUS CKX 41) and fluorescence microscope (model: OLYMPUS IX71) were purchased from Olympus (Tokyo, Japan). The fluorescence quantitative polymerase chain reaction (PCR) instrument (model: 7900HT) was purchased from Applied Biosystems (Waltham, MA, USA). The flow cytometer (model: BD LSR II) was obtained from Becton Dickinson (Franklin, NJ, USA).

### Cell Culture

The TJ905 human glioma cells were derived from glioblastoma multiforme that had been excised during surgical operation and were provided by the Neural Oncology Institute of Tianjin Medical University and maintained as monolayers in DMEM containing 10% FBS with 100 U/mL penicillin and 100 µg/mL streptomycin at 37°C in 5% CO_2_ and 95% humidified air. The glioma stem cells were cultivated in neural stem cell (NSC) medium, containing DMEM/F12 with high glucose, 20 ng/mL bFGF, 10 ng/mL LIF, B27 (1×), and maintained at 37°C in 5% CO_2_ and 95% air. The medium was replaced every 3–4 days. After 8–10 days of cultivation, neurospheres were observed (11). Glioblastoma U251 cell line was provided by China Center for Typical Culture Collection (CCTCC) (Wuhan, China) and cultured under the same condition as TJ905 cells.

### Cell Sorting for CD133^+^ Cells

TJ905 and U251 cells were collected and cultivated separately in NSC medium as described earlier for 10 days. CD133^+^ cells were separated from the neurospheres using a magnetic activated cell sorting technique. Following cell sorting, the CD133^+^ cells were cultivated in NSC medium.

### Nestin, GFAP, and β-Tubulin Immunofluorescence Staining of Glioma Stem Cells

Well-growing cell spheres were transferred to slides coated with polylysine and placed in six-well plates. After drying at 37°C, the slides were washed with phosphate-buffered saline (PBS) three times to remove the residual medium. The cells were fixed with 4% paraformaldehyde for 30 min at room temperature and then washed with PBS three times. Subsequently, the cells were blocked with 5% goat serum at 37°C for 30 min, a nestin antibody was added, and the cells were placed in a humidified box overnight. Then, cells were washed with PBS and incubated with a fluorescein isothiocyanate-conjugated monoclonal goat anti-rabbit IgG secondary antibody for 30 min at 37°C. In addition, a negative control in which the primary antibody was replaced with PBS was performed. Images of stained slides were captured under a fluorescence microscope after three washes with PBS. The immunofluorescence staining procedure for GFAP and β-tubulin was the same as the procedure used for nestin (12). Nestin, GFAP, and β-tubulin were obtained from Santa Cruz Biotechnology (Santa Cruz, CA, USA) and the dilution was 1:200 for each primary antibody, while the dilution for secondary antibody was 1:50.

### The Identification of Glioma Stem Cells

Well-growing cell spheres were transferred to slides coated with polylysine, placed in a six-well plate, and cultured in DMEM containing 10% FBS for 5–7 days. When cell spheres with pseudopodia emerged and exhibited a morphological change, immunofluorescence staining for nestin, GFAP, and β-tubulin was performed.

### Lentivirus Transfection

The procedure was performed using the method reported in our previous studies (13–15). TJ905 cells were seeded into 12-well plates at a density of 5 × 10^5^ cells/well and cultured for 12 h. Subsequently, the cells were infected with the lentivirus at an MOI of 20 and appropriate volume of polybrene was added, ensuring a final concentration of polybrene to 5–10 µg/mL. Then, the cell culture medium containing the lentivirus was replaced 24 h after infection and the expression of GFP was observed 3–5 days later under a fluorescence microscope to calculate the cell transfection rate. The cells in the well with highest transfection rate were passaged. The infection processes used for glioma stem cells were similar to the process described earlier. Prior to transfection, cell spheres were dissociated into single cell suspensions and the MOI was 40. Groups examined in the experiments were as follows:

Group A:TJ905 cells transfected with the Livin-shRNA
Group B:TJ905 cells transfected with the negative-shRNA
Group C:Monolayer culture of TJ905 glioma cells
Group D:Glioma stem cells transfected with the Livin-shRNA
Group E:Glioma stem cells transfected with the negative-shRNA
Group F:Monolayer culture of TJ905 glioma stem cells.

### CCK-8 Assay

Glioma cells and glioma stem cells were seeded into 96-well plates at density of 10^5^ cells/well and cultured for 24 h. Then, the medium was replaced with 100 µL of medium containing various concentrations of TMZ (0, 25, 50, 100, 200, and 400 µmol/L). TMZ was substituted with the CCK-8 solution 48 h later, and the plate was incubated for 1.5 h at 37°C. The absorbance was measured at 490 nm. The experiments were repeated in duplicate.

### Real-time Quantitative PCR Analysis

The experiment was performed as described in our previous studies (16, 17). At 48 h after the TMZ intervention, total RNA was extracted from the cells in each group using TRIzol reagent. Reverse transcription was performed using a one-step RT-PCR kit. PCR was undertaken using the real-time PCR Master Mix reagent kit, according to the manufacturer’s protocol. Primer Premier Software (version 5.0) was used to design primers for the fluorescent qPCR. The sequences of the primers are listed below. The 2^ΔΔCt^ method was used to calculate the relative expression levels.

### Homo-Livin

5′-GCTGTCAGTTCCTGCTCCGGTC-3′
5′-CAGGGGCTGCGTCTTCCGGTTC-3′

### Homo-Caspase-3

5′-GAAGCGAATCAATGGACTCTGG-3′
5′-GTTTGCTGCATCGACATCTGTAC-3′

### Homo-Caspase 7

5′-CCTCGTTTGTACCGTCCCTCTT-3′
5′-GGCATCTTTGTCTGTTCCGTTTC-3′

### Homo-Caspase 9

5′-GCTCTTCCTTTGTTCATCTCCTG-3′
5′-GCACCGACATCACCAAATCCTC-3′

### Flow Cytometry Analysis to Detect Changes in the Cell Cycle

Groups are the same as described earlier, and cells were dissociated into single cell suspensions. Cells were incubated overnight at 4°C with 70% alcohol and washed with ice-cold PBS. Then, 500 µL of PI staining fluid was added and the cells were incubated at 37°C for 30 min and placed on ice until detection. Finally, the data were analyzed using the Byo software.

### Statistical Analysis

The statistical analysis was performed using the methods reported in our previous studies (18, 19). All experimental results are presented as mean ± SEM. Statistical analyses were performed using the SPSS 12.0 software. Statistical analyses were performed using Student’s *t*-test and one-way analysis of variance. A *P* value of less than 0.05 was considered significant.

## Results

### Separation and Identification of Glioma Stem Cells

**Figure 1:**
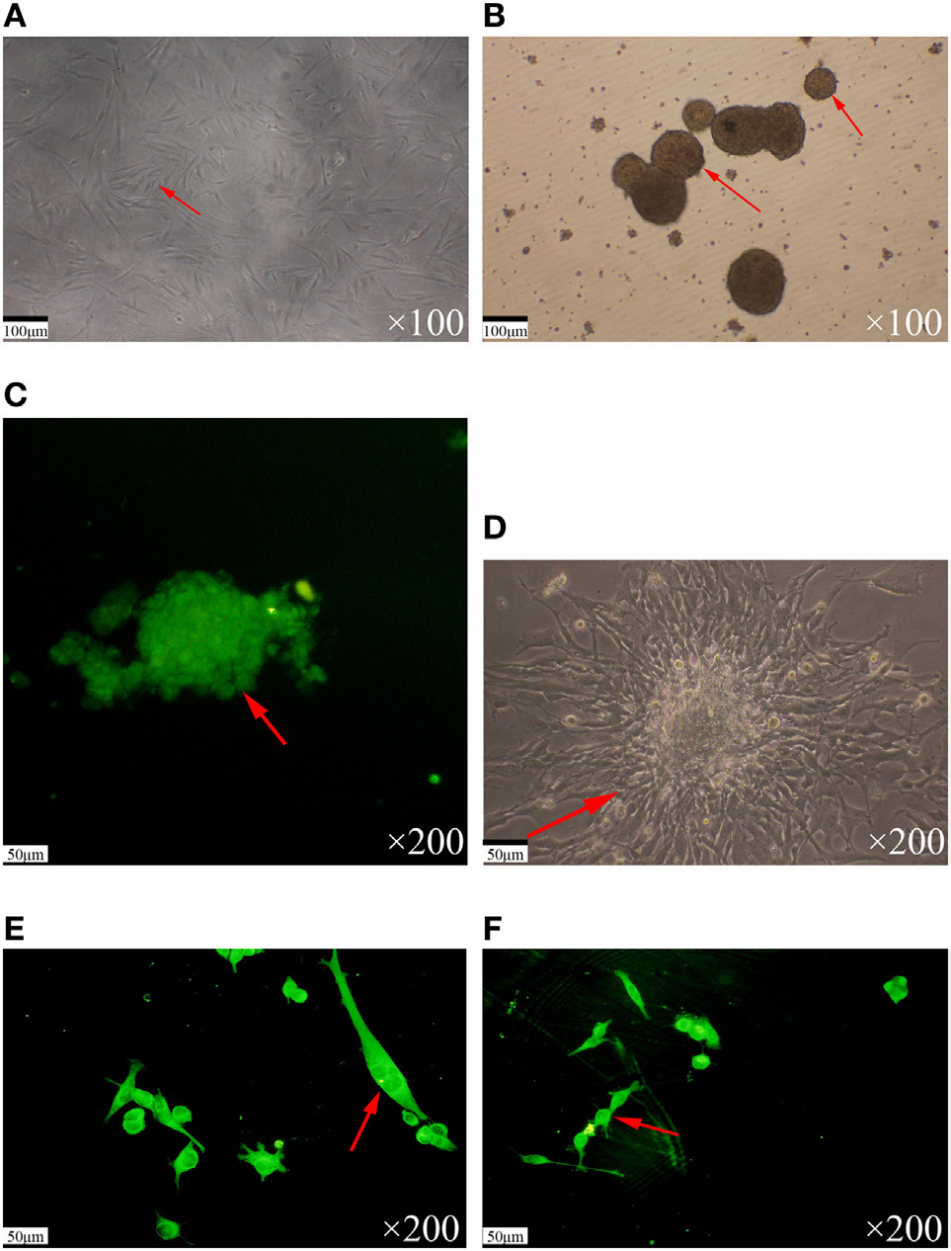
Separation and identification of glioma stem cells. **(A)** The TJ905 cells were cultured in serum-free medium. The cells grew closely with few pseudopodia. **(B)** The TJ905 stem cells grew together to spheres in suspension after 3–4 days. **(C)** Immunofluorescence staining showed that the nestin immunostaining for spheres was strongly positive. **(D)** After culture in medium containing 10% fetal bovine serum, the spheres differentiated, appearing with many pseudopodia and showing many kinds of irregular shapes, such as triangle, round, and star shapes. **(E)** The GFAP immunostaining for differentiated glioma stem cells was strongly positive and nestin staining was negative. **(F)** The β-tubulin immunostaining for differentiated glioma stem cells was strongly positive.

Using serum-free culture and the magnetic activated cell sorting method, the TJ905 glioma stem cells were successfully sorted from TJ905 cells. The proportion of cancer stem cells was approximately 0.64–0.91%. The TJ905 cells cultured in serum-free media adhered to the wall of the vessel, showing a shuttle shape or triangular and irregular forms. The cells were arranged closely and few pseudopodia were observed (Figure 1A). Cells were successfully separated into CD133^+^ and CD133^−^ populations using immunomagnetic beads. The CD133^+^ cells were cultured in stem cell medium, and most were suspended in the medium. After 3–4 days, most of the suspended cells had formed spheres and grew in suspension (Figure 1B). After the stem cells were cultured for 7 days, immunofluorescence staining for nestin, GFAP, and β-tubulin was performed. Nestin exhibited strong positive immunoreactivity (Figure 1C), whereas GFAP and β-tubulin exhibited negative immunoreactivity. Furthermore, after medium containing 10% FBS was used to culture the CD133^+^ cells, the spheres promptly adhered to the wall of the vessel and differentiated, presenting many pseudopodia and many irregular shapes, such as triangle, round, and star shapes (Figure 1D). After 10 days of differentiation, immunofluorescence staining for nestin, GFAP, and β-tubulin was conducted. Immunofluorescence staining for nestin was negative, but immunofluorescence staining for GFAP was strongly positive (Figure 1E), and β-tubulin staining was positive (Figure 1F), indicating that glioma stem cells had differentiated.

### Establishment of the Livin Transfection Model in TJ905 Cells and Stem Cells

**Figure 2:**
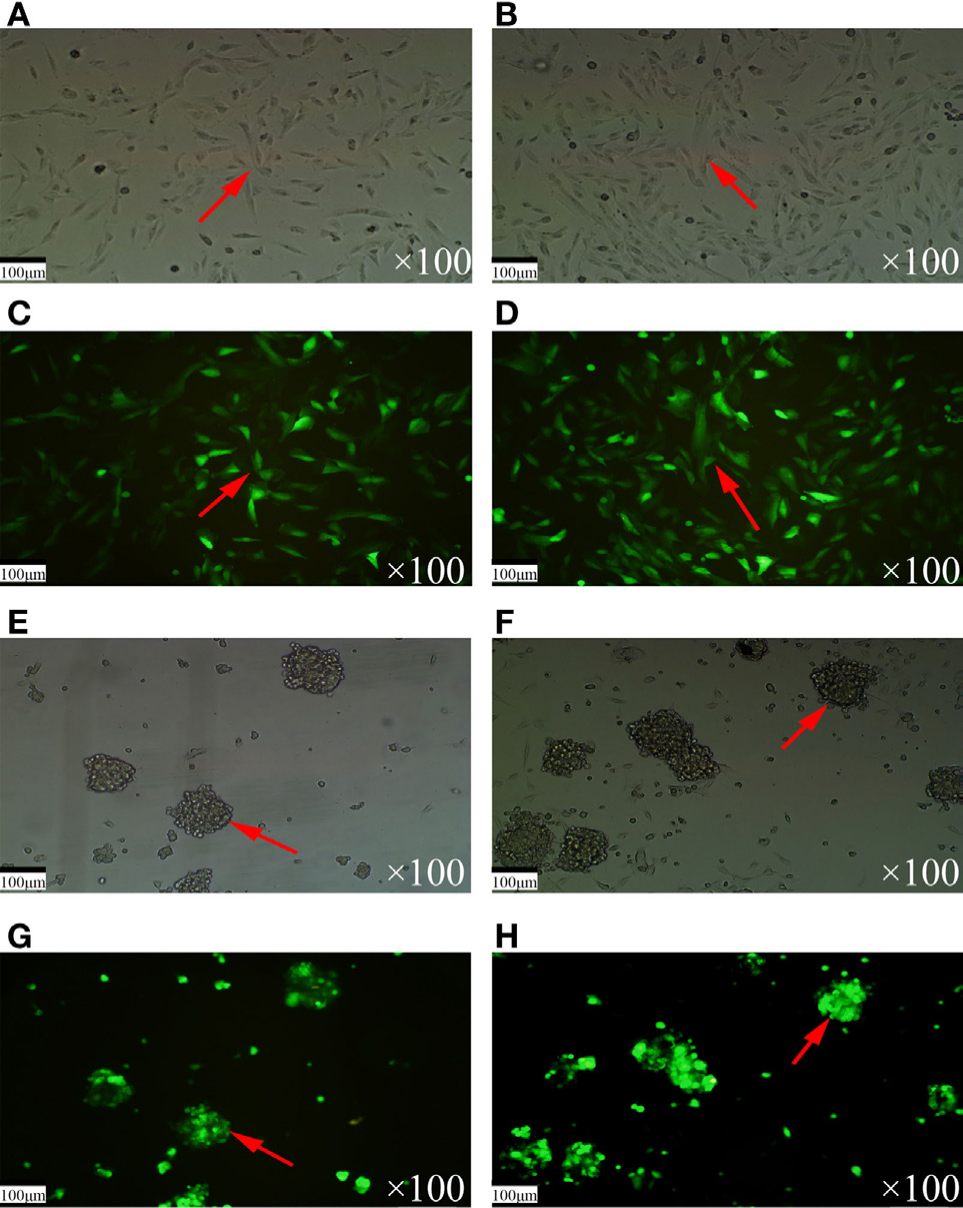
The morphology of TJ905 cells and stem cells under optical and fluorescence microscopy. **(A,B)** The TJ905 cells appeared normal under optical microscopy. **(C,D)** The TJ905 cells were luminous under fluorescence microscopy. **(E,F)** The TJ905 stem cells in suspended growth state under optical microscopy. **(G,H)** The TJ905 stem cells were luminous under fluorescence microscopy.

A Livin gene transfection model was the key feature of this study and the foundation of this experiment. Therefore, we selected a lentiviral vector to establish the Livin transfection model. A lentiviral vector expressing the Livin gene or the empty vector was successfully transfected into TJ905 cells and stem cells to establish the cell models. Because the selected plasmid contains the green fluorescent protein gene, successfully transfected cells stably expressed green fluorescent protein and exhibited green fluorescence under the fluorescent microscope. TJ905 cells exhibited a normal morphology and adhered to the wall of the vessel under an ordinary optical microscope (Figures 2A,B) and displayed the corresponding cell morphology under the fluorescence microscope (Figures 2C,D). Glioma stem cells exhibited suspended growth with a normal morphology under an ordinary optical microscope (Figures 2E,F) and the corresponding cell morphology under a fluorescence microscope (Figures 2G,H).

### Detection of Cell Proliferation Activity Using the CCK-8 Assay

**Figure 3:**
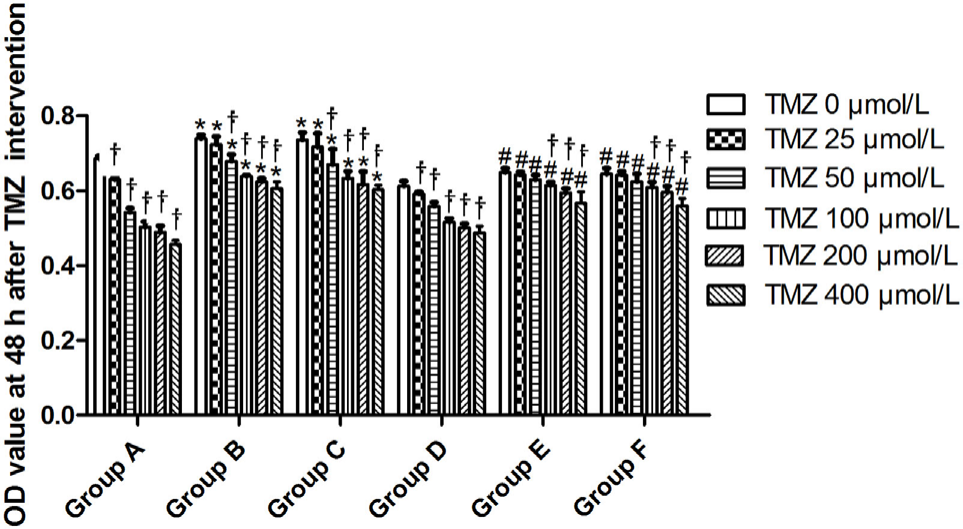
The change of OD value at 48 h after temozolomide (TMZ) intervention. *P < 0.05, compared with Group A in the same concentration; ^†^*P* < 0.05 compared with the concentration of 0 µmol/L; ^#^*P* < 0.05 compared with Group D in the same concentration. Group A: TJ905 cells transfected with Livin-shRNA, Group B: TJ905 cells transfected with negative-shRNA, Group C: the monolayer culture of TJ905 glioma cells, Group D: glioma stem cells transfected with Livin-shRNA, Group E: glioma stem cells transfected with negative-shRNA, and Group F: the monolayer culture of TJ905 glioma stem cells.

The proliferation rate of TJ905 cells was higher than derived glioma stem cells. TMZ significantly inhibited the proliferation of TJ905 cells and stem cells in a dose-dependent manner, displaying stronger inhibition toward TJ905 cells. The Livin-shRNA markedly inhibited the proliferation of TJ905 cells and stem cells, which was also more distinct in TJ905 cells (Figure 3).

### Sensitivity of Glioma Cells and Glioma Stem Cells to TMZ before and after Livin-shRNA Transfection

TJ905 cells transfected with the Livin-shRNA were more sensitive to TMZ than cells transfected with the negative-shRNA and controls, resulting in reduced drug resistance and indicating that the Livin gene might play a crucial role in the resistance mechanism of TJ905 glioma cells. Glioma stem cells transfected with the Livin-shRNA showed no significant changes in sensitivity to TMZ compared with cells transfected with the negative-shRNA and controls, indicating that the resistance mechanisms differed between TJ905 glioma cells and stem cells. In view of the lower levels of the Livin mRNA observed in glioma stem cells, the roles of the Livin gene in the resistance of glioma cells and stem cells to chemotherapy differed.

### Real-time Quantitative PCR Analysis of the Expression of Related Genes

**Figure 4:**
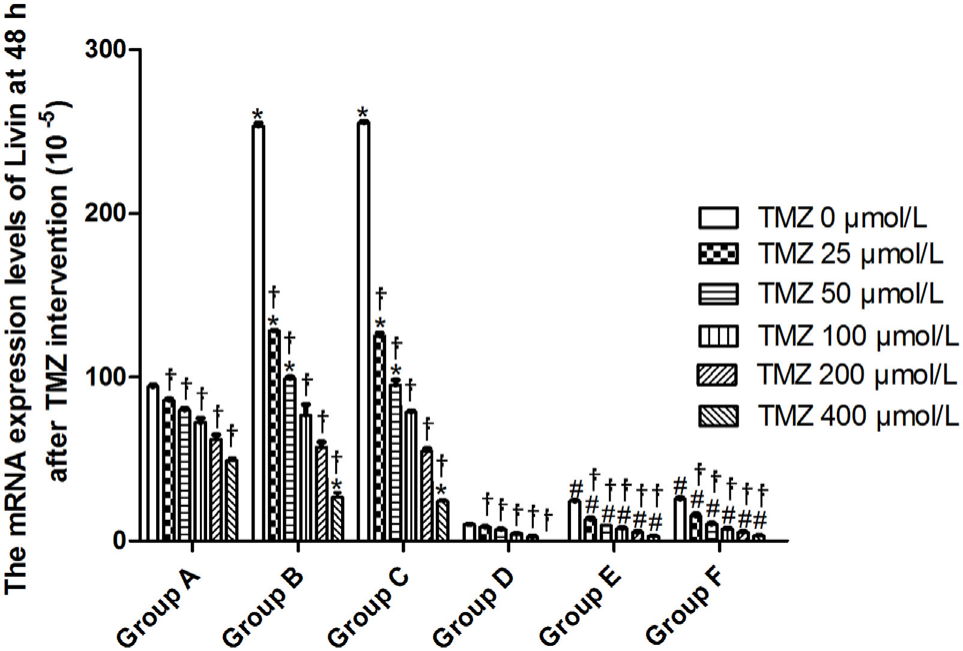
The mRNA expression levels of Livin at 48 h after temozolomide (TMZ) intervention (10^−5^). *P < 0.05, compared with Group A in the same concentration; ^†^*P* < 0.05 compared with the concentration of 0 µmol/L; ^#^*P* < 0.05 compared with Group D in the same concentration. Group A: TJ905 cells transfected with Livin-shRNA, Group B: TJ905 cells transfected with negative-shRNA, Group C: the monolayer culture of TJ905 glioma cells, Group D: glioma stem cells transfected with Livin-shRNA, Group E: glioma stem cells transfected with negative-shRNA, and Group F: the monolayer culture of TJ905 glioma stem cells.

**Figure 5:**
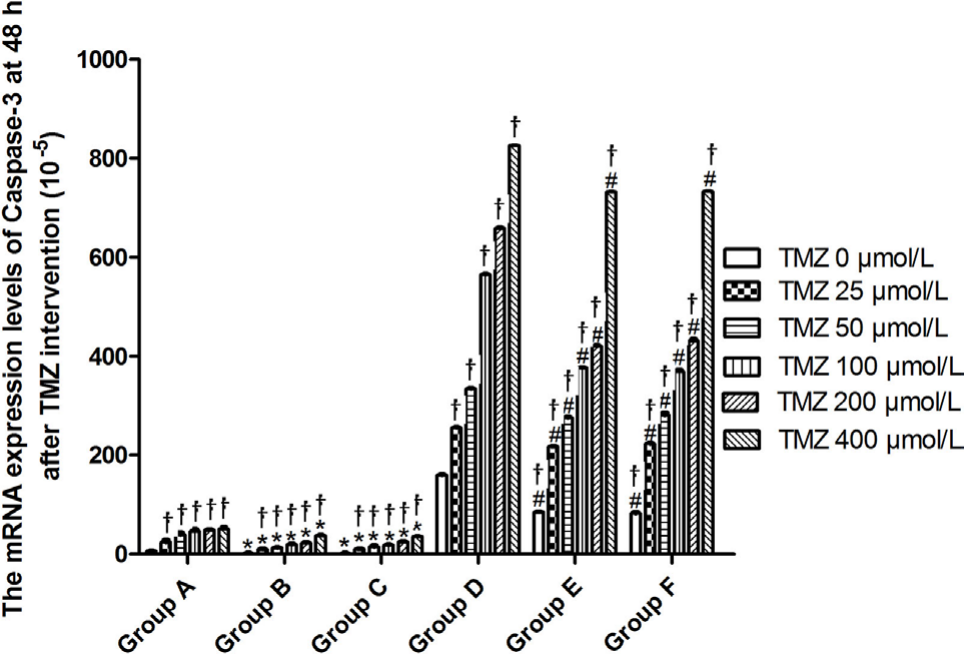
The mRNA expression levels of Caspase-3 at 48 h after temozolomide (TMZ) intervention (10^−5^). *P < 0.05, compared with Group A in the same concentration; ^†^*P* < 0.05 compared with the concentration of 0 µmol/L; ^#^*P* < 0.05 compared with Group D in the same concentration. Group A: TJ905 cells transfected with Livin-shRNA, Group B: TJ905 cells transfected with negative-shRNA, Group C: the monolayer culture of TJ905 glioma cells, Group D: glioma stem cells transfected with Livin-shRNA, Group E: glioma stem cells transfected with negative-shRNA, and Group F: the monolayer culture of TJ905 glioma stem cells.

**Figure 6:**
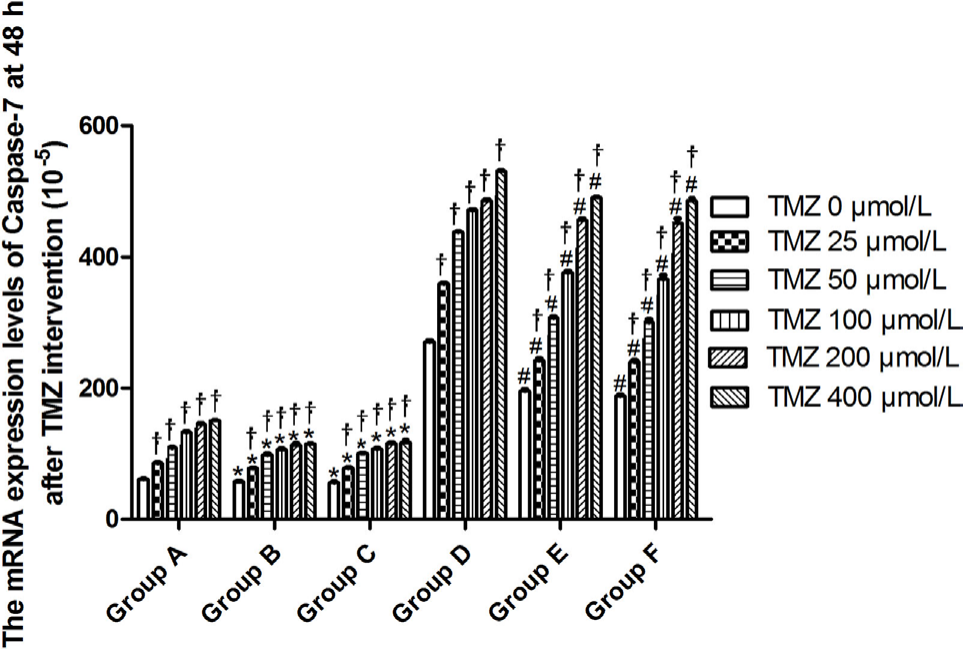
The mRNA expression levels of Caspase-7 at 48 h after temozolomide (TMZ) intervention (10^−5^). *P < 0.05, compared with Group A in the same concentration; ^†^*P* < 0.05 compared with the concentration of 0 µmol/L; ^#^*P* < 0.05 compared with Group D in the same concentration. Group A: TJ905 cells transfected with Livin-shRNA, Group B: TJ905 cells transfected with negative-shRNA, Group C: the monolayer culture of TJ905 glioma cells, Group D: glioma stem cells transfected with Livin-shRNA, Group E: glioma stem cells transfected with negative-shRNA, and Group F: the monolayer culture of TJ905 glioma stem cells.

**Figure 7:**
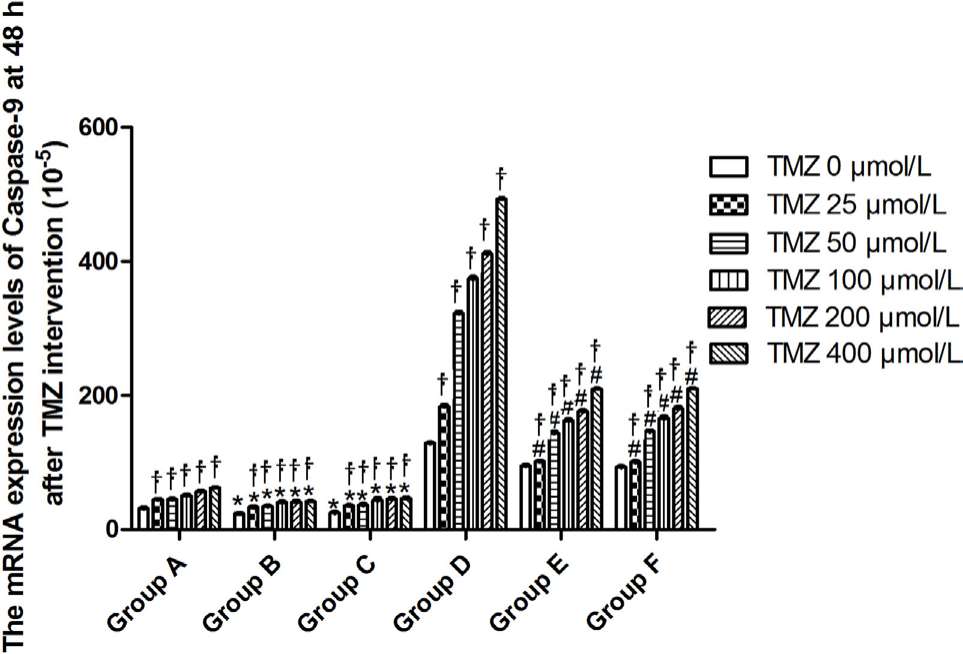
The mRNA expression levels of Caspase-9 at 48 h after temozolomide (TMZ) intervention (10^−5^). *P < 0.05, compared with Group A in the same concentration; ^†^*P* < 0.05 compared with the concentration of 0 µmol/L; ^#^*P* < 0.05 compared with Group D in the same concentration. Group A: TJ905 cells transfected with Livin-shRNA, Group B: TJ905 cells transfected with negative-shRNA, Group C: the monolayer culture of TJ905 glioma cells, Group D: glioma stem cells transfected with Livin-shRNA, Group E: glioma stem cells transfected with negative-shRNA, and Group F: the monolayer culture of TJ905 glioma stem cells.

**Figure 8:**
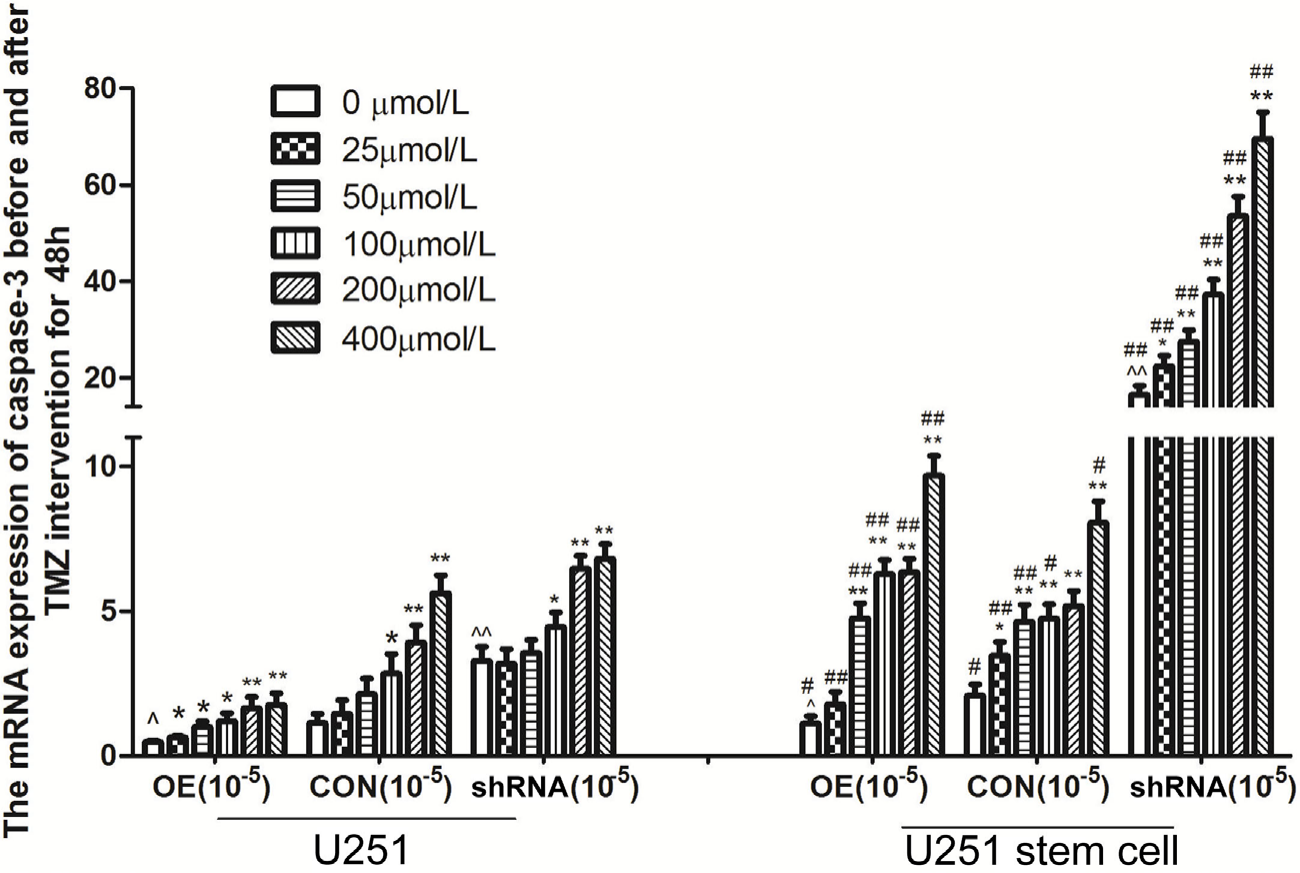
The mRNA expression of caspase-3 before and after temozolomide (TMZ) intervention for 48 h in U251 cells and stem cells. Data presented as mean ± SD. *P < 0.05 and **P < 0.01, compared to the same cells with 0 µmol/L; ^*P* < 0.05 and ^^*P* < 0.01, OE compared to CON or siRNA compared to CON in same cell with 0 µmol/L, *^#^P* < 0.05 and *^##^P* < 0.01, CSC compared to ACC as the same drug concentration in OE, CON and siRNA, respectively. U251; U251 stem cell; OE, overexpression Livin group; CON, control group; shRNA: shRNA-Livin group.

**Figure 9:**
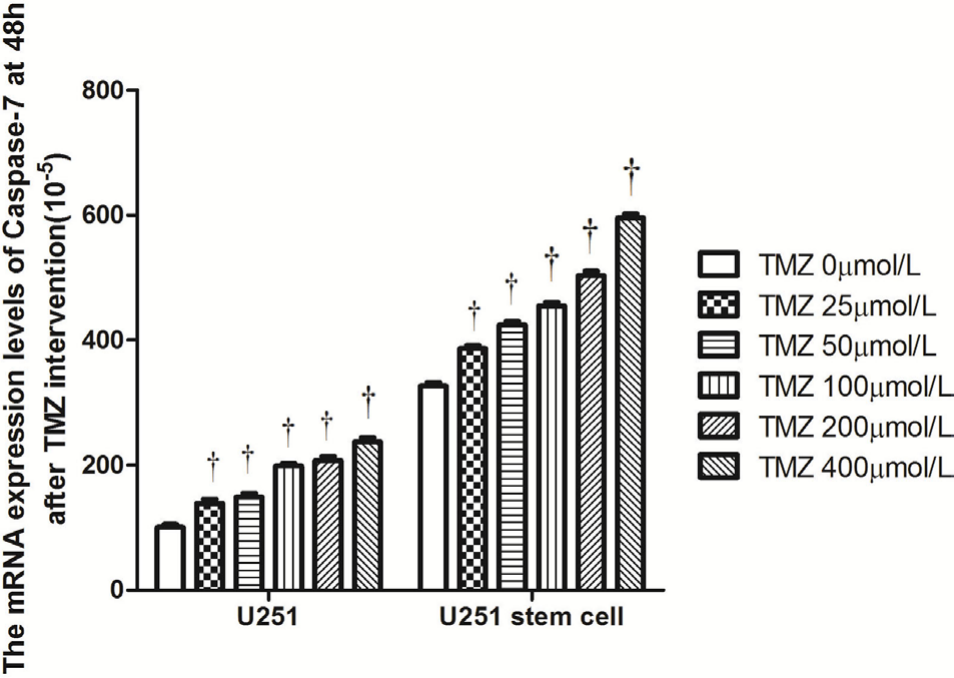
The mRNA expression levels of Caspase-7 at 48 h after temozolomide (TMZ) intervention (10^−5^) in U251 cells and stem cells. ^†^*P* < 0.05 compared to the same cells with 0 µmol/L.

Livin mRNA expression was significantly higher in TJ905 cells than in glioma stem cells. TMZ inhibited Livin mRNA expression in TJ905 cells and stem cells and induces the expression of the Caspase-3, -7, and -9 mRNAs. Livin mRNA expression was reduced in TJ905 glioma cells and stem cells following transfection with the Livin-shRNA, and the expression of the Caspase-3, -7, and -9 mRNAs was also significantly increased (Figures 4–7). We repeated the experiments of Caspase-3 and -7 expressions in U251 cells and stem cells and found the results were similar to those obtained with TJ905 cells and stem cells (Figures 8 and 9).

### Flow Cytometry Analysis of the Changes in the Cell Cycle

**Figure 10:**
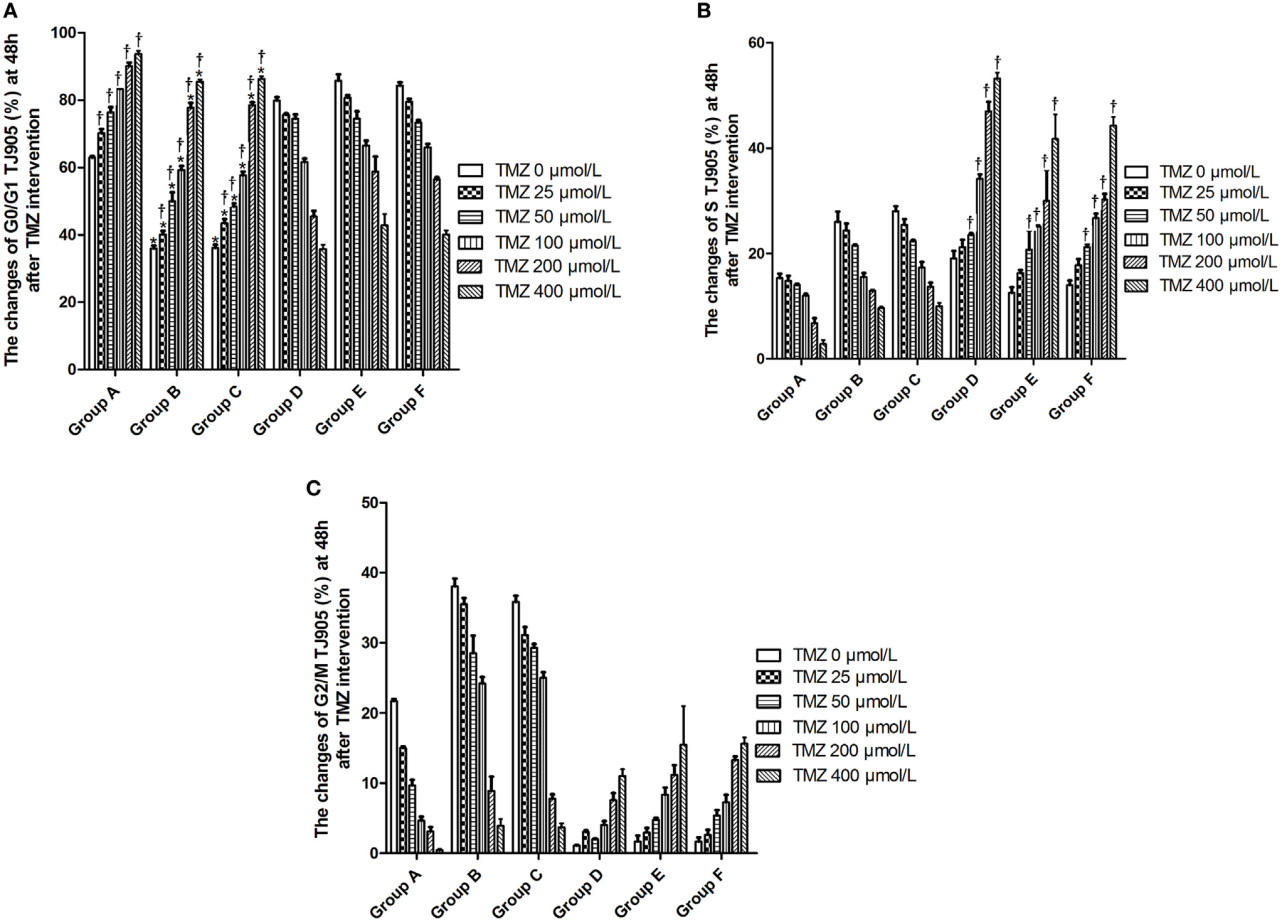
The changes of cell cycle at 48 h after temozolomide (TMZ) with different concentration interfering with TJ905 glioma cells (%). **(A)** G0/G1 phase; **(B)** S phase; **(C)** G2/M phase. *P < 0.05, compared with Group A in the same concentration; ^†^*P* < 0.05 compared with the concentration of 0 µmol/L; ^#^*P* < 0.05 compared with Group D in the same concentration. Group A: TJ905 cells transfected with Livin-shRNA, Group B: TJ905 cells transfected with negative-shRNA, Group C: the monolayer culture of TJ905 glioma cells, Group D: glioma stem cells transfected with Livin-shRNA, Group E: glioma stem cells transfected with negative-shRNA, and Group F: the monolayer culture of TJ905 glioma stem cells.

Temozolomide arrested the growth of TJ905 glioma cells in G0/G1 phase and glioma stem cells in S phase in a dose-dependent manner. The Livin-shRNA arrested the growth of TJ905 glioma cell in G0/G1 phase but showed no significant impact on the cell cycle of glioma stem cells (Figure 10).

## Discussion

Although maximally effective therapies with surgery, chemotherapy, and radiation are performed in clinical practice, glioma, particularly glioblastoma, remains the most common and devastating primary brain tumor (20). Recent studies have identified glioma stem cells in glioblastoma and showed that these cells likely play a key role in resistance to conventional therapies, as well as recurrence (21). Glioma stem cells are capable of self-renewal and differentiation and *de novo* tumor formation for xenograft implantation. Furthermore, these cells possess unique signaling pathways to promote tumorigenesis and vascular formation (22). Therefore, glioma stem cell growth and proliferation must be controlled to eradicate the recurrence of glioma after the administration of combined therapies.

In our study, TJ905 tumor stem cells were isolated with the magnetic activated cell sorting method and the characteristics of the cancer stem cells were identified using immunofluorescence staining for nestin, β-tubulin, and GFAP. Immunofluorescence staining for nestin but not GFAP and β-tubulin was observed in the cancer stem cells, which contrasts with the results observed in cancer cells. After cancer stem cells had been induced with normal culture medium, the results of the immunofluorescence staining were the same as TJ905 cells, which also confirmed the successful isolation of glioma stem cells (23, 24).

Chemotherapy resistance is the main focus of treatments for malignant glioma (25). The resistance of glioma remains difficult to resolve in clinical practice. Two types of cells were treated with TMZ to examine whether glioma stem cells are equipped with stronger capability for drug resistance than glioma cells. TMZ effectively inhibited the proliferation of TJ905 glioma cells and stem cells in a dose-dependent manner and had a greater effect on TJ905 cells, which was consistent with other reports and revealed the presence of different resistant mechanism in the two cell types. Livin is a novel member of the IAP family. Its overexpression in TJ905 glioma cells and stem cells reduced cell apoptosis; thus, Livin exerts an anti-tumor effect. The changes in Livin expression induced by TMZ suggest that Livin may contribute to chemotherapy drug resistance.

Recently, shRNA transfection has emerged as a powerful technique, allowing transcriptional silencing of target genes in cells (26). The mechanism of shRNA is not precisely transcriptional silencing. The mRNAs are synthesized but are unable to be effectively translated to proteins (26). Therefore, we adopted the shRNA transfection technique to explore the role of the Livin gene in drug resistance and proliferation in TJ905 glioma cells and stem cells. After successfully transfecting the shRNA, the Livin-shRNA was stably expressed in the cells and significantly inhibited Livin mRNA expression in TJ905 glioma cells and stem cells.

Temozolomide is included in the standard care for patients with glioblastoma multiforme after maximal safe surgical resection followed by adjuvant partial brain radiation (27). The therapeutic benefit of TMZ depends on its capability to alkylate/methylate DNA, which damages the DNA and triggers the death of tumor cells (28). However, the interaction between TMZ and the Livin gene is unclear. In our study, TMZ inhibited Livin mRNA expression in TJ905 cells and stem cells and induced the expression of the Caspase-3, -7, and -9 mRNAs, thereby inhibiting cell proliferation to exert its anti-tumor effect. The Livin gene may be upstream of Caspase-3, -7, and -9. Low Livin mRNA expression induced the expression of the Caspase-3, -7, and -9 mRNAs, thus promoting cell apoptosis. In addition, we tested the mRNA expression of Caspase-3 and -7 in U251 glioma and derived glioma stem cells and found similar results, which strengthened the findings obtained from TJ905 glioma cells and derived stem cells. TMZ may well exert its anti-tumor effect by inhibiting Livin mRNA expression. TJ905 cells transfected with the Livin-shRNA were more sensitive to TMZ than cells transfected with the Negative-shRNA and controls, indicating that the Livin gene may play a crucial role in the drug resistance mechanism of TJ905 cells. However, for glioma stem cells, the Livin-shRNA did not induce a significant change TMZ sensitivity compared to the Negative-ShRNA and controls, illustrating that the resistance mechanisms of TJ905 glioma cells and stem cells differed. Because the Livin mRNA was expressed at lower levels in glioma stem cells, glioma stem cells were equipped with stronger resistance to TMZ than TJ905 cells. The Livin gene is not the only factor involved in drug resistance in glioma stem cells.

Temozolomide and the Livin-shRNA effectively inhibited the proliferation of TJ905 cells and stem cells, and the effects were more apparent on TJ905 cells, suggesting that the Livin gene partially promoted cell proliferation. Regarding the influence on the cell cycle, TMZ and the Livin-shRNA consistently exerted an inhibitory effect on glioma cells by inducing cell cycle arrest in G0/G1 phase. In contrast, TMZ exerted an inhibitory effect on glioma stem cell growth by inducing cell cycle arrest in S phase. However, the Livin-shRNA did not have a significant impact on the cell cycle of glioma stem cells, indicating that the Livin gene played different roles in drug resistance and the cell cycle of TJ905 cells and stem cells. TMZ likely inhibited the cell cycle of glioma stem cells through another mechanism.

In conclusion, glioma cells and derived stem cells showed strong resistance to TMZ. The Livin gene played different roles in drug resistance and the cell cycle of TJ905 glioma cells and stem cells. The Livin gene might play a vital role in the drug resistance of TJ905 cells, but it is not the only factor involved in drug resistance in glioma stem cells. In addition, some findings were confirmed in U251 cells and stem cells, which consolidated the conclusions of our study. As well, MGMT promoter methylation status plays an impartment role in glioblastoma and was not reported in the TJ905 cells; further studies will focus on how Livin interacts with MGMT promoter methylation and replicating the experiments in other primary patient-derived glioblastoma cell lines and so on. The mechanism of resistance to chemotherapeutic agents requires further study of new therapeutic targets for glioma in the future.

## Author Contributions

FJ conceived and designed the experiment, analyzed and interpreted the data. G-KH and HZ participated in immunofluorescence staining assay and contributed to data acquisition. RZ and G-HL isolated tumor stem cells and transfected Livin-shRNA into cells. SF and X-YQ did CCK-8 assay and flow cytometry. L-SK, Q-MN, and H-RL performed real-time PCR. LZ conceived the study and revised the manuscript critically for important intellectual content.

## Conflict of Interest Statement

The authors declare that the research was conducted in the absence of any commercial or financial relationships that could be construed as a potential conflict of interest.
